# Altered force generation and cell-to-cell contractile imbalance in hypertrophic cardiomyopathy

**DOI:** 10.1007/s00424-019-02260-9

**Published:** 2019-02-11

**Authors:** Theresia Kraft, Judith Montag

**Affiliations:** Molecular and Cell Physiology, Hannover Medical School, Carl-Neuberg-Str. 1, 30625 Hannover, Germany

**Keywords:** Hypertrophic cardiomyopathy, Contractile imbalance, Allelic imbalance, Burst-like transcription, Weak binding states

## Abstract

Hypertrophic cardiomyopathy (HCM) is mainly caused by mutations in sarcomeric proteins. Thirty to forty percent of identified mutations are found in the ventricular myosin heavy chain (β-MyHC). A common mechanism explaining how numerous mutations in several different proteins induce a similar HCM-phenotype is unclear. It was proposed that HCM-mutations cause hypercontractility, which for some mutations is thought to result from mutation-induced unlocking of myosin heads from a so-called super-relaxed state (SRX). The SRX was suggested to be related to the “interacting head motif,” i.e., pairs of myosin heads folded back onto their S2-region. Here, we address these structural states of myosin in context of earlier work on weak binding cross-bridges. However, not all HCM-mutations cause hypercontractility and/or are involved in the interacting head motif. But most likely, all mutations alter the force generating mechanism, yet in different ways, possibly including inhibition of SRX. Such functional—hyper- and hypocontractile—changes are the basis of our previously proposed concept stating that contractile imbalance due to unequal fractions of mutated and wildtype protein among individual cardiomyocytes over time will induce cardiomyocyte disarray and fibrosis, hallmarks of HCM. Studying β-MyHC-mutations, we found substantial contractile variability from cardiomyocyte to cardiomyocyte within a patient’s myocardium, much higher than in controls. This was paralleled by a similarly variable fraction of mutant *MYH7*-mRNA (cell-to-cell allelic imbalance), due to random, burst-like transcription, independent for mutant and wildtype *MYH7*-alleles. Evidence suggests that HCM-mutations in other sarcomeric proteins follow the same disease mechanism.

## Introduction

### Hypertrophic cardiomyopathy

Hypertrophic cardiomyopathy (HCM) is a genetic cardiac disease with an incidence of 1:500 [75]. Recent reevaluation of the prevalence of disease-causing mutations indicates an even higher incidence of 1:200 [101]. The clinical onset of HCM is highly variable; it ranges from a nearly asymptomatic disease course to arrhythmias, syncopes, and the development of heart failure. Also, sudden cardiac death in young and mostly asymptomatic athletes is characteristic for HCM [73]. HCM is characterized by asymmetric hypertrophy of the left ventricle and/or the interventricular septum that is not caused by other pathologies such as hypertension or aortic stenosis. In the myocardium of HCM-patients, cardiomyocytes and myofibrils are often highly disordered, and cardiomyocytes show variable size and shape. Cell-to-cell contacts are partially disrupted and myocytes are oriented in different directions [27, 72]. This so-called myocyte and myofibrillar disarray is regarded as hallmark of HCM and its degree appears associated with the severity of disease progression [24, 31, 74, 118].

### Mutations that lead to HCM

To date, more than 1500 mutations in up to 26 genes have been linked to HCM [71]. However, several mutations need to be reevaluated whether they are truly disease-causing according to the ACMG (American College of Genetics and Genomics) guidelines [96]. A recent study that compared sequence data from 3267 individuals diagnosed with HCM and 60,706 reference samples from the Exome Aggregation Consortium (ExAC) shows that several variants that were considered disease-causing are in fact quite common in the reference population and thereby may not be pathogenic [123]. Interestingly, about 90% of all mutation-positive HCM-patients carry mutations in only four out of the 26 genes. These are the *MYH7*-gene encoding for the ventricular β-myosin heavy chain (β-MyHC), the *MYBPC3*-gene encoding for cardiac myosin-binding protein C (cMyBP-C), and *TNNT2* and *TNNI3* encoding for cardiac troponin T and troponin I, respectively [71, 95, 123]. Among these, *MYH7* and *MYBPC3* are the most commonly affected genes with approximately 30–50% of genotyped patients each; the ratios vary between different cohorts [29, 36, 48, 53, 79, 95, 123]. In rare cases (3–5%), which are often associated with a severe phenotype, two mutations either in the same gene (double heterozygosity) or in different genes (compound heterozygosity) are found [95, 115].

### β-MyHC mutations in HCM

**Fig. 1:**
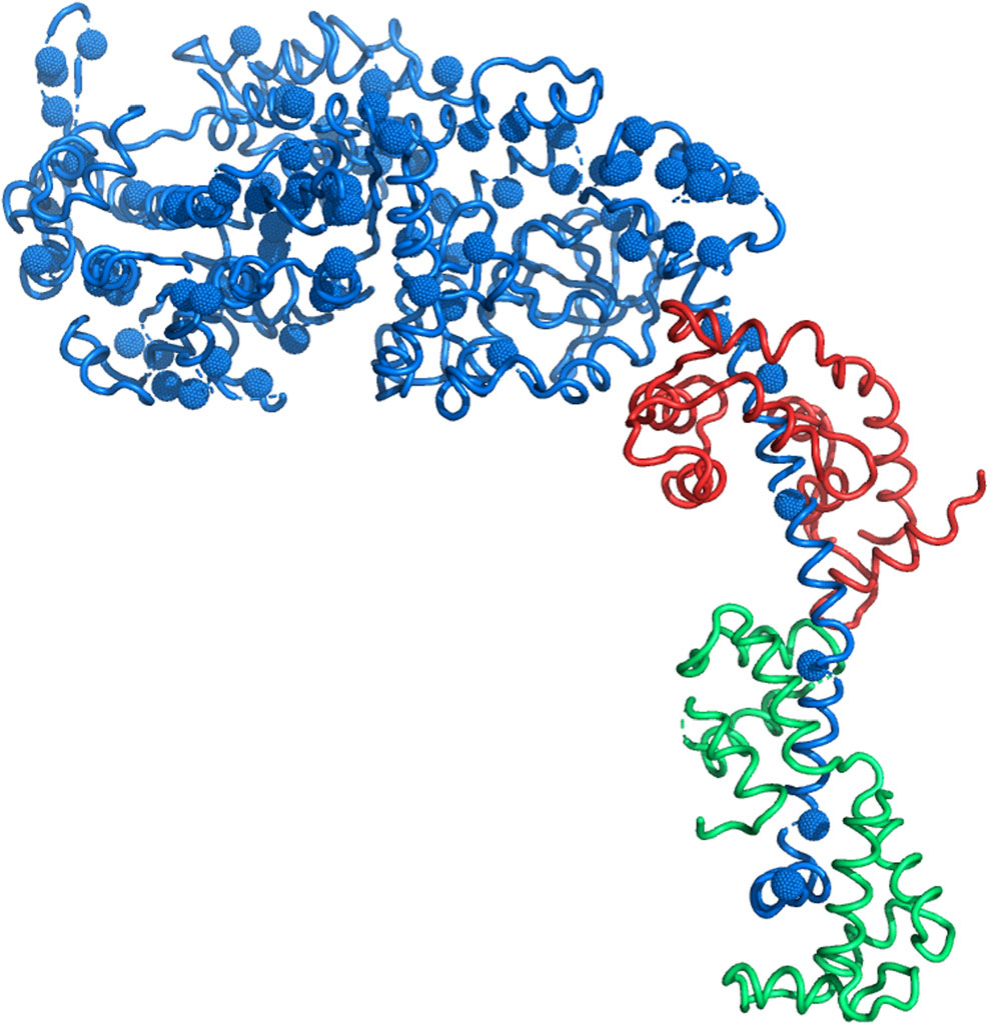
HCM-mutations in the S-1 region of β-MyHC. Structure of the S-1 part of β-MyHC (blue) protein based on Rayment and colleagues [92] with mutations validated as HCM-causing by Walsh and co-workers [123]. The light chains are indicated in red (ELC) and green (RLC). Note that the mutations are distributed all over S-1

In this review, we mainly focus on mutations in β-MyHC (*MYH7*) which is the predominant myosin isoform in ventricular sarcomeres [78]. To date, more than 400 HCM-associated mutations have been described in *MYH7*, of which more than 95% are missense mutations [20, 122]. Most β-MyHC mutations are clustered between residues 181 and 937 (Fig. 1). These residues form the myosin head domain (S-1), which includes the motor domain with the actin binding site and the ATPase site, the converter domain and the ELC-binding region of the lever arm, and a small part of the S-2 portion of the myosin rod [83, 123]. Approximately 20% are located in the coiled coil region (S-2) that forms the thick filament [20]. HCM-related β-MyHC missense mutations have been found to alter several different parameters of active contraction such as isometric force levels, cross-bridge cycling kinetics, acto-myosin ATPase-activity, shortening velocity, calcium sensitivity of force generation, and relaxation properties of the sarcomeres (for reviews see [17, 83]).

Active contraction in skeletal and cardiac muscle is based on ATP-driven elastic deformation of myosin heads resulting in generation of force and sarcomere shortening [42, 45, 46]. The cross-bridge cycle in calcium-activated striated muscle can be described by a two-state model (Huxley-Brenner two-state model) [11, 13, 16, 43]. During force generation the acto-myosin cross-bridges cycle between two groups of states, the force generating, strong binding states with high actin affinity and the non-force generating, weak binding states with low actin affinity. In the weak binding states, ATP is hydrolyzed to ADP and P_i_ but both products remain bound to the cross-bridge. Biochemical studies and experiments on muscle fibers showed that weak binding cross-bridges rapidly bind and unbind actin [14, 15, 18, 107, 125]. This weak binding is not sufficient to generate force [12, 19, 37]. Yet, weak attachment of myosin to actin is an essential intermediate for the transition into the force-generating states [16, 59]. The electrostatic, non-stereospecific weak interaction of myosin with actin occurs at specific sites on actin that are different from the tropomyosin-regulated strong binding sites [60, 63, 70, 128]. The so-called power stroke is associated with calcium binding to the troponin-tropomyosin complex, phosphate release from myosin, and a transition of the myosin heads from a non-stereospecific weak-binding conformation to a stereospecific, strong-binding conformation. Completion of the power stroke is coupled to release of ADP and rapid rebinding of ATP (for reviews see [34, 41, 104]).

Since mutations in β-MyHC are located essentially in all different functional subdomains of the molecule and particularly of the myosin head domain (Fig. 1), it is not surprising that these mutations affect the described mechanism of force generation in various ways [17, 83].

## Hypotheses on the pathomechanisms in HCM

### Poison peptide mechanism, haploinsufficiency, and disease severity

The effects of β-MyHC missense mutations on sarcomere function suggested that the mutated myosin is incorporated into the sarcomeres and that the normal mechanism of force generation is disturbed [9]. This dominant negative “poison peptide effect” of mutated β-MyHC was supported by studies showing altered sarcomere function and yet undisturbed packing of myofibrils or myofilaments, indicating normal incorporation of the mutated myosin into the thick filaments and the sarcomeres [58].

Interestingly, the level of severity of HCM seems to be associated with the relative abundance of mutated vs. wildtype protein in the myocardium. The usually more severe course of disease in homozygous patients as compared to heterozygous relatives was assumed to be due to the higher amount of mutant protein in the homozygous patients [87, 94]. This assumption was supported by findings in heterozygous HCM patients where in several cases, a benign disease course was associated with a low relative fraction of mutant vs. wildtype mRNA and protein [7, 26, 35, 39, 81, 86, 111]. The unexpected deviation from a 50:50 ratio of mutant vs. wildtype transcript and protein in myocardial samples was called “tissue level allelic imbalance” [81]. In addition, downregulation of mutant mRNA and protein in an HCM-mouse model could reduce HCM-pathology such as hypertrophy and myocyte disarray [49]. However, in some HCM-patients and mouse models low levels of mutated β-MyHC were found associated with early disease onset and severe phenotype. This indicates that not only the relative fraction of mutant β-MyHC but also the location and the functional alterations caused by the mutations themselves contribute to the severity of the disease [39, 81, 121].

Also, for several other disorders, phenotypic severity has been directly linked to the increased expression of alleles with disease-causing mutations. High fractions of transcripts from the allele encoding for a mutant K_v_7.1 potassium channel (loss-of-function) seems to cause a more severe form of long QT-syndrome [4]. In malignant hyperthermia patients, allelic imbalance of the ryanodine receptor gene was determined which might underlie the variable penetrance of the disease [38]. Allelic imbalance resulting in increased expression of the mutant allele can also lead to onset of a recessive disorder in heterozygous patients, as shown for a causative mutation in Zellweger Spectrum Disorder [30]. In summary, in several diseases including HCM, the increased expression of the disease-causing allele seems to contribute to severity, pathogenic phenotype and the clinical onset of the respective disorder.

The poison peptide hypothesis is well-established particularly for β-MyHC-mutations but also for HCM missense mutations in other sarcomeric proteins. However, some mutations—especially truncating nonsense and frameshift mutations in *MYBPC3*—lead to the expression of C-terminally truncated isoforms that are usually not incorporated in the sarcomeres [98]. Here, no poison peptide effect but most likely the lack of functional cMyBP-C—so-called haploinsufficiency—seems to lead to malfunction of the sarcomeres [133], including reduced maximal force generation and secondary altered calcium sensitivity due to changes in myofilament protein phosphorylation [113] (for review see [100]).

But how can various mutations in distinct functional areas of one gene—and even more in several different genes—either through a poison peptide mechanism or haploinsufficiency lead to a similar disease phenotype? In the next sections, current hypotheses on HCM pathogenesis will be addressed with a main focus on mutations in *MYH7*.

### The hypercontractility hypothesis

Already, in early studies on HCM, both “hypercontractility” and cardiac hypertrophy, as well as “hypocontractility” followed by hypertrophy to compensate for the impairment, were described as pathophysiological mechanisms leading to HCM [9]. One current hypothesis based on clinical evidence and experimental findings at the molecular level is that HCM-mutations cause hypercontractility, while DCM mutations lead to hypocontractility (for review see [6, 106]). A number of HCM mutations in sarcomeric proteins showed increased calcium sensitivity, higher maximum force generation, and increased ATPase activity. This was associated with higher tension cost, leading to defects in cellular and myocardial energetics and reduced energy reserves, which seem to be common in HCM. Yet, functional effects of several HCM-mutations are incompatible with the “hypercontractility hypothesis.” Results show that instead, contractility and calcium sensitivity can be increased, decreased or unaltered in HCM [57, 61, 80, 114, 119]; for review see [83]. For example, the converter mutations R719W and R723G cause increased maximum force generation and decreased calcium sensitivity in slow skeletal and cardiac muscle of HCM-patients [57, 61]. In recombinant human-truncated myosin-S-1 fragments, reduced intrinsic force and unaltered ATPase-activity were observed [54]. Mutation R453C showed reduced ATPase activity [8, 103] and reduced force in heart tissues engineered from human pluripotent stem cell-cardiomyocytes [84]. For the most intensively studied β-MyHC mutation R403Q conflicting results on the increase or decrease of ATPase-activity, force generation, velocity, etc. have been reported [83, 126]. One explanation of these inconsistent results might reside in the respective study setup; the use of either native, expressed, full length, or truncated, α-, or β-MyHC of different species seems to influence the effect.

### Structural states of the thick filament and HCM

During recent years, two novel aspects of myosin structure and ATPase-function were revealed which may be relevant for HCM pathology: the interacting head motif and the so-called super-relaxed state, a state of myosin in relaxed muscle with much reduced ATPase activity. Here, we discuss both concepts in the light of previous studies on the conformation of thick filaments and myosin heads in relaxed muscle.

#### Weak binding cross-bridge states and myosin layer lines

It has been known for a long time that myosin heads can assume several different conformations including a helically ordered structure. In X-ray diffraction, the helically ordered structure of the myosin heads near the thick filament surface typically gives rise to strong myosin layer lines (MLL) and related meridional reflections [44]. The intensity of the MLLs in skeletal and cardiac muscle strongly depends on temperature [69, 70, 128], on the ligand bound or the biochemical state of myosin heads [34, 129–131]. The helical order as indicated by strong MLLs requires a closed conformation of the myosin heads (“closed” as defined by Geeves and Holmes and others, where switch 2 of S1 stabilizes the γ-phosphate in an intermediate conformation [32, 34, 102]).

Early on, the coexistence of ordered and disordered myosin head populations was postulated [89]. It was observed that lowering the temperature reduces the MLL intensities and increases diffuse scattering, indicating a reduction of the fraction of helically ordered cross-bridges and increasing disorder. Interestingly, lowering ionic strength (which increases the fraction of cross-bridges weakly bound to actin [12, 14, 132]) essentially does not affect the MLLs [60, 69, 128]. X-ray diffraction data from relaxed striated muscle at different temperatures and ionic strengths together with model calculations provided evidence for three populations of myosin head conformations which are in dynamic equilibrium: helically ordered myosin heads situated close to the backbone of the thick filament, disordered detached heads and disordered heads that are weakly attached to actin [70, 128].

Not only in skeletal muscle but also in cardiac muscle, evidence for cross-bridges weakly attached to actin was provided [130]. Since weak attachment to actin enables myosin heads to sense the activation status of the thin filament, most likely, also in cardiac muscle, weak binding cross-bridge states are essential intermediates on the path to force generation.

#### The super-relaxed state

Single nucleotide turnover experiments on permeabilized, relaxed skeletal muscle fibers and strips from rabbit ventricular muscle revealed nucleotide release rates from myosin with a relatively fast and an extremely slow component [40, 108]. The slow component of the basal ATP turnover was attributed to a so-called super-relaxed state (SRX) of myosin with a much reduced metabolic rate. The authors assumed that in cardiac muscle (not in skeletal muscle), a subset of myosin molecules remain in the SRX even during activation of the muscle and thus may slightly reduce the total metabolic rate of working cardiac muscle. Correlations between X-ray diffraction studies and the SRX experiments suggested that SRX myosin heads contribute to the helically ordered population of myosin heads discussed above [108]. The SRX could thus contribute to the strong MLLs in relaxed skeletal and cardiac muscle in addition to previously described other myosin/thick filament conformations [66, 131].

Recently, changes in MLLs and meridional reflections of myosin during activation of frog skeletal muscle fibers [68] and cardiac preparations from rat [93] suggested that stress on the thick filament at increasing loads of the sarcomere may lead to an increase in force by recruitment/unlocking of myosin heads from the helically ordered population on the filament backbone. The authors assume that these might be SRX heads and conclude that the thick filament might act as additional regulatory mechanosensor in skeletal and cardiac muscle. This could provide a fast mechanism for recruiting myosin heads from the SRX for force generation at high load to allow adjustment of end-diastolic volume-dependent systolic force from heart beat to heart beat known as the Frank-Starling-Mechanism [68]. Further studies are warranted to clarify whether this idea is consistent with well-characterized kinetics of force development and redevelopment in different muscle types.

Other mechanisms for unlocking of helically ordered myosin heads have been suggested early on for tarantula thick filaments [22] and also for mouse cardiac muscle including phosphorylation of the RLC which results in more disordered myosin heads [21]. In skinned trabeculae of rat ventricle, RLC phosphorylation was found to change the myosin conformation from helical order (as suggested for the SRX) towards a more perpendicular orientation relative to the backbone. This was associated with increased calcium sensitivity and force [52]. Interestingly, the orientation of the RLC changed not only upon phosphorylation of the RLC, but also through calcium activation and at longer sarcomere length [52]. This suggests that myosin heads in the helically ordered state (or SRX) can be activated under several conditions which may include both, short term (thick filament stress) and long term (phosphorylation) modulations of cardiac contraction [47].

#### The interacting head motif

It was suggested that structurally the SRX corresponds to myosin heads forming the so-called interacting head motif on the thick filament backbone (IHM; for review see [76]). Electron microscopy and 3D reconstructions of two-headed myosin of smooth muscle thick filaments and of myosin-regulated tarantula-striated muscle with unphosphorylated RLC indicated an asymmetric interaction of the two heads of myosin dimers [124, 127]. The actin-binding domain of the so-called blocked head was found linked to the converter and the essential light chain of the second “free” head. Both myosin heads were folded back onto their own coiled-coil S2-part. The intra- and intermolecular interactions of the two heads in this motif are thought to inhibit binding to actin and to result in much reduced basal ATP turnover, as seen earlier for the helically ordered myosin heads in scallop myosin with extremely slow ATP-release [120]. This conformation appears to be stabilized via ionic interactions [51, 97]. Further studies using 3D single-particle analysis on isolated myosin filaments confirmed this myosin motif in different muscle types, including mouse and human cardiac muscle in the relaxed state and in the absence of actin [135] [3].

In smooth muscle, the folded-back state most likely represents inhibited myosin that can be activated by RLC phosphorylation [124], representing the major regulatory mechanism of smooth muscle contraction. In skeletal and cardiac muscle, calcium binding to troponin C and the resulting activation of the thin filament regulate cross-bridge cycle activity and thus force generation. Nevertheless, structural evidence exists that also in skeletal muscle, RLC-phosphorylation can modulate the conformation of myosin heads and thus acto-myosin interaction [65]. Furthermore, for thick filaments isolated from mouse cardiac muscle, it was shown that cMyBP-C may also contribute to the ordered structure of the myosin filament, and that phosphorylation of cMyBP-C results in more disordered filaments [55, 56].

#### SRX, interacting head motif, myosin mesa hypothesis, and hypercontractility in HCM

Overall, the evidence is not unequivocal whether the interacting head motif indeed is related to the SRX and to the helically ordered myosin heads that give rise to the strong MLLs. The helical order of the thick filaments may as well directly result from the closed myosin head conformation as defined by Geeves and Holmes [34]. Quantitative considerations suggested that the disorder/order equilibrium of myosin heads in relaxed muscle is not determined by release/formation of the interacting head motif [131]. An interesting recent study on purified human myosin constructs with different length of the proximal S2-region and in the absence of actin addressed the question whether the SRX in the muscle fibers is related to the interacting head motif observed in structural studies on isolated thick filaments [5]. The authors found that the fraction of myosin heads with SRX-like basal ATPase rates increased with lowering ionic strength. With mavacamten, a cardiac myosin inhibitor, basal ATP turnover of myosin in solution was reduced, suggesting stabilization of the SRX state [5, 136]. Electron microscopy on human myosin constructs using cross-linkable mavacamten revealed a substantial fraction of a folded head state, which could represent the interacting head motif [5]. In studies on porcine cardiac muscle strips, mavacamten reduced active tension by half and strongly enhanced the MLLs in relaxation and activation, indicating a larger fraction of helically ordered cross-bridges [5].

Yet, further work is needed to provide direct evidence about putative contributions of the interacting head motif to the SRX and of both to MLLs. From many previous studies on acto-myosin cross-bridge structure and turnover kinetics in vitro and in sarcomeres, several predictions arise that can be tested to further characterize the SRX and the interacting head motif in cardiac and skeletal muscle, including the recruitment of myosin heads from the interacting head motif under physiological conditions which must be very rapid. High-resolution structural studies on intact sarcomeres are desirable to reveal the occupancy of this state/structural conformation of myosin in situ.

How could SRX and interacting head motif be linked to β-MyHC mutations and HCM? It was hypothesized that HCM-mutations in β-MyHC which are located in or close to structures involved in the formation of the interacting head motif interfere with back-folding of the myosin heads [83]. This could decrease the total number of myosin heads in the SRX, resulting in higher basal ATPase rate and possibly increased contractility of cardiomyocytes in HCM and may even impair relaxation, thus affecting diastolic and systolic function in HCM. Spudich observed that several mutations in the β-MyHC motor domain map to a particular mesa-shaped surface area which appears in a pre-power stroke conformation of S1 [105]. This area contains an arginine-rich, positively charged region, which may interact with cMyBP-C and titin and also with the proximal S2 region of same the myosin dimer, thus strengthening the interacting head motif [85, 105, 106]. The “myosin mesa hypothesis” suggested that HCM mutations in the myosin mesa and in the converter alter the charge of these regions, and possibly in related domains of cMyBP-C. This would diminish the ionic S1-S2 interactions and release the myosin heads from an inhibited state, resulting in hypercontractility. In cardiac samples from HCM-patients with mutations in cMyBP-C, evidence for destabilization of myosin heads in the SRX was found [77]. Structural analysis of locations and charge changes of HCM-associated variants in β-MyHC and myosin light chains with respect to the interacting head motif conformation also suggested that several mutations could impair the formation of the interactions and thus could reduce the myosin head fraction in the SRX [1, 97]. However, β-MyHC-mutations not in the mesa or converter did not affect S1-S2 interactions and thus were considered unlikely to contribute to disturbed formation of the SRX [85]. Also, several mutations in the myosin mesa, converter and proximal S2-region actually increase the number of positively charged amino acids [112] which could keep more myosin heads in the folded-back state and thus may not cause hypercontractility.

## Unequal allelic expression of wildtype and mutated protein from cell to cell and the contractile imbalance hypothesis

### Cardiomyocytes from HCM-patients reveal large functional variability from cell to cell

Recently, our group suggested the “contractile imbalance hypothesis” as novel concept for the development of typical HCM-features like cellular disarray and interstitial fibrosis. It is based on the observation that single cardiomyocytes from the very same HCM-patient generated highly different force levels at identical Ca^++^ - concentrations [62].

**Fig. 2:**
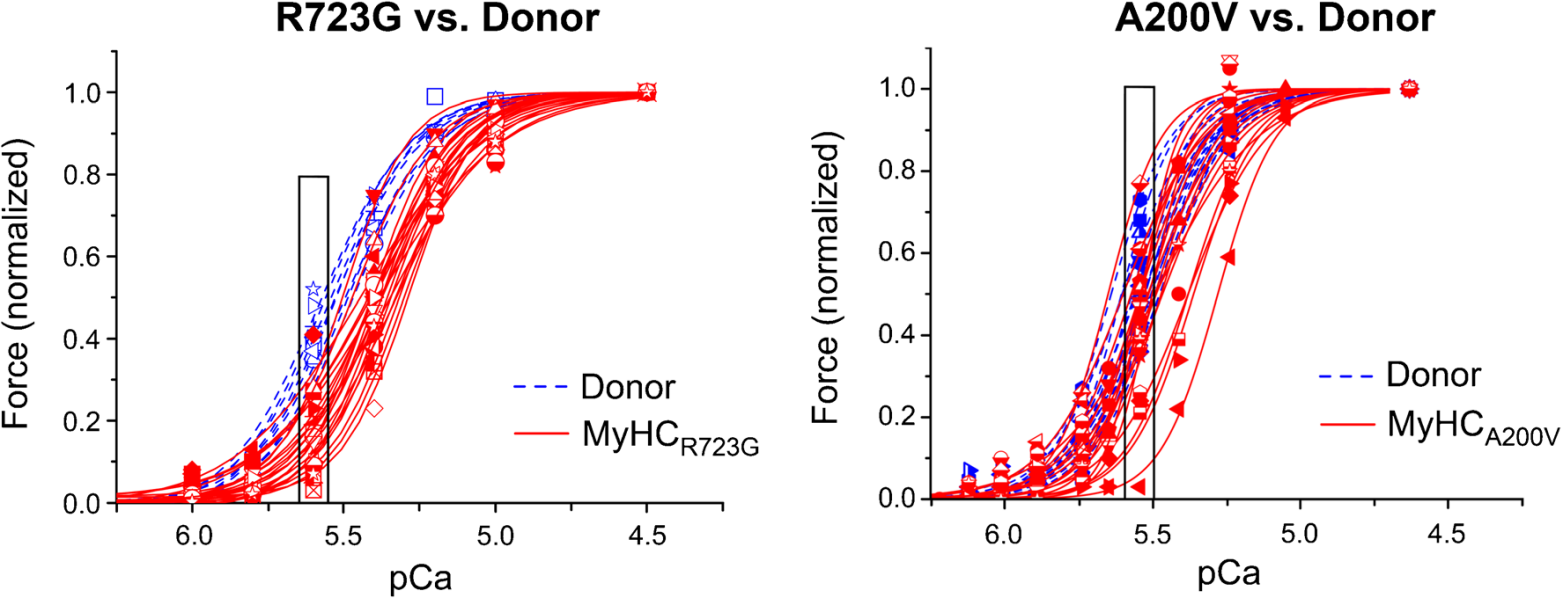
Contractile heterogeneity of individual cardiomyocytes from HCM-patients compared to donor cardiomyocytes. Single cardiomyocytes were isolated from frozen heart tissue of HCM-patients (red) with the mutation R723G (left) or A200V (right), respectively, and from donor individuals (blue) as controls. Cardiomyocytes were permeabilized, and after adjustment of phosphorylation levels [57, 61, 80, 114, 119], they were subjected to different calcium concentrations and the respective force generation was measured. Depicted are the forces of individual left ventricular cardiomyocytes at different calcium concentrations (force-pCa-relations), normalized to maximum force. Each symbol and curve represents a different individual cell. The boxes at physiological calcium concentration highlight the much larger variance in force generation among individual cardiomyocytes from the patients compared to controls. Figure reprinted from [82] and modified, with permission from Frontiers

We have shown that calcium-dependent force generation of isolated, permeabilized single cardiomyocytes from HCM-patients with two different β-MyHC-mutations (A200V and R723G) vary substantially from cell to cell for each HCM-patient, significantly more than for donor cardiomyocytes that were used as controls (Fig. 2). Some patient cardiomyocytes showed calcium sensitivity comparable to controls, whereas others showed substantially reduced calcium sensitivity. At physiological calcium concentrations, relative force generation of individual cardiomyocytes differed 10–20-fold comparing the weakest with the strongest cardiomyocyte with mutations R723G or A200V, respectively. In contrast, in both controls, forces at the same calcium concentration varied only about 1.5-fold [82].

We expect that a similar contractile imbalance will develop if a mutation alters other parameters of cardiomyocyte function such as shortening velocity or relaxation properties [57]. Furthermore, we have preliminary evidence that also for HCM-cardiomyocytes with mutations in cMyBP-C [2] or in cTnI (unpublished) such functional imbalance from cell to cell exists.

Our findings suggest that force generation of individual cardiomyocytes during systole is highly variable in the myocardium of HCM-patients. The functional heterogeneity will cause contractile imbalance where stronger cardiomyocytes may over-contract while weaker cardiomyocytes may be over-stretched. This effect may disrupt the myocardial network and lead to the HCM-associated myocyte disarray. Moreover, increased stretching of myocardial cells induces the release of TGF-β, angiotensin II, and endothelin-1 [99, 116] and the expression of hypertrophic markers in cultured neonatal cardiomyocytes [117]. An HCM-mouse model showed that the—presumably mutation-induced—increased expression of TGF-β was directly associated with the activation of pro-fibrotic pathways and with hypertrophic remodeling [109]. Accordingly, we assume that contractile imbalance between cardiomyocytes of HCM-patients not only induces myocyte disarray but also the release of TGF-β and other cytokines, thus triggering fibrosis and hypertrophy [17].

### Cell-to-cell allelic imbalance among cardiomyocytes and *M. soleus* fibers of HCM-patients as underlying cause for functional imbalance

In earlier studies on slow *M. soleus* fibers from HCM patients which express β-MyHC, we also observed a large functional variability among individual fibers. Calcium sensitivity ranged from normal to highly shifted for mutations R719W and R723G, while for fibers with mutation I736T, highly variable incomplete relaxation was found [57]. We asked whether the functional heterogeneity could be due to unequal fractions of mutant and wildtype β-MyHC in the individual fibers. Relative quantification of *MYH7*-mRNA from individual *M. soleus* fibers with mutation R723G revealed a large variability of the fraction of R723G-mRNA ranging from 100 to less than 20% [17]. Previously, highly variable fractions of mutated protein had been determined in skeletal muscle fibers with mutation R403Q [70].

**Fig. 3:**
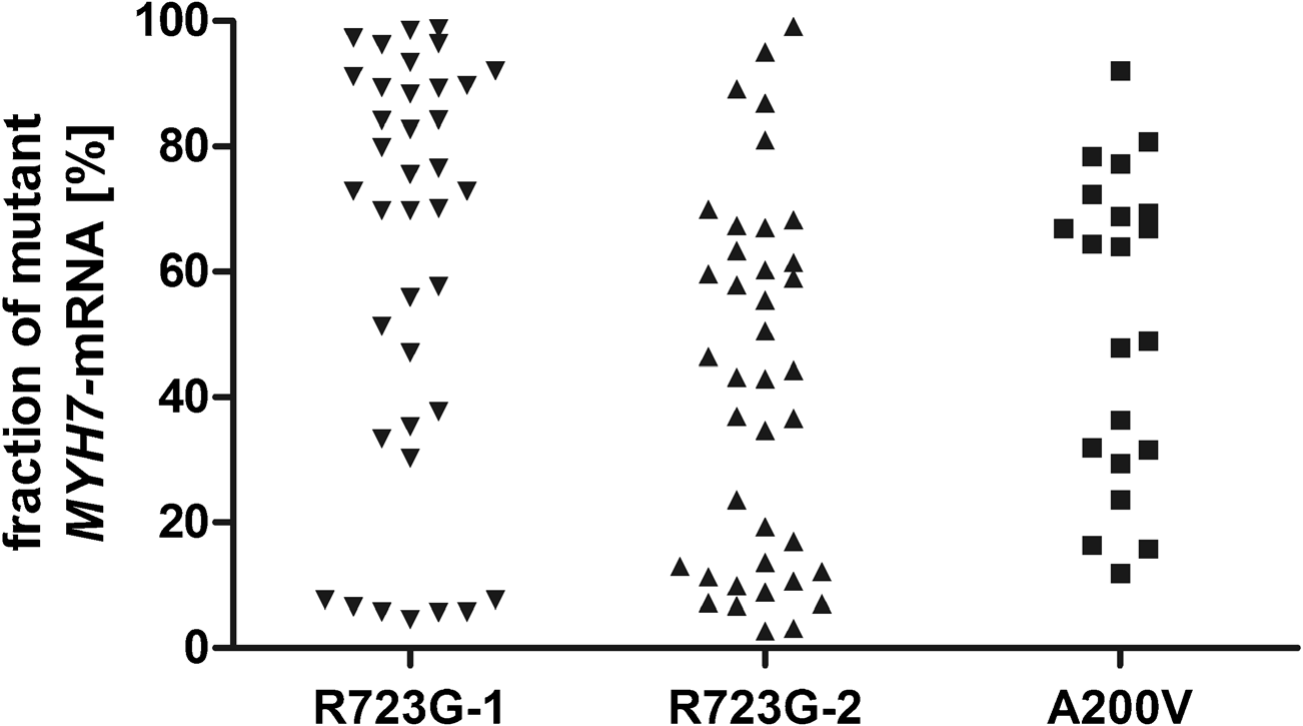
Cell-to-cell allelic imbalance of *MYH7*-mRNA in three HCM patients. Individual cells were isolated from sections of cardiac tissue via laser capture microdissection. Cells were lysed and the *MYH7*-mRNA was amplified by single cell RT-PCR. The fractions of mutant vs. wildtype transcript were determined by densitometric analysis of allele-specific restriction digests. Depicted are the fractions of mutant *MYH7*-mRNA in individual cardiomyocytes from three different HCM-patients (R723G-1, R723G-2, and A200V). Each dot represents one cardiomyocyte

The unequal fractions of mutated and wildtype *MYH7*-mRNA in *M. soleus* fibers suggested that such cell-to-cell allelic imbalance might also underlie the functional imbalance in cardiomyocytes. We adapted the method and quantified the relative expression of wildtype vs. mutant *MYH7*-mRNA in single cardiomyocytes of the same myocardial samples (R723G-1 and A200V) in which we had determined the contractile imbalance [62, 82]. In addition, we analyzed a further patient with mutation R723G (R723G-2). We found cardiomyocytes with almost exclusively wildtype *MYH7*-mRNA, with different fractions of mRNA from both alleles, and with almost exclusively mutant *MYH7*-mRNA in each patient (Fig. 3), indicating cell-to-cell allelic imbalance. Control experiments showed that not only variance in mutant and wildtype *MYH7*-mRNA fractions but also in function from cell to cell was much larger than the experimental error [62, 82]. We assume that the functional heterogeneity is due to the unequal expression of mutated and wildtype β-MyHC from cell to cell [62, 82]. So far, no direct correlation of mutated vs. wildtype β-MyHC protein fractions and cardiomyocyte function is possible. This would require functional analysis followed by highly sensitive quantitative mass-spectrometric β-MyHC analysis of the same single cardiomyocytes. However, mathematical simulations that took into account published rate constants for mRNA and protein life times, and the effect of variable fractions of mutant β-MyHC on calcium sensitivity strengthened our conclusion [62, 82].

### Burst-like transcription as underlying mechanism of cell-to-cell allelic imbalance

Which mechanism could trigger this striking heterogeneity of *MYH7*-allele expression among individual cardiomyocytes? The traditional model of continuous gene expression of both alleles would most likely lead to a rather homogeneous allelic expression pattern from cell to cell and is expected to result in a Poisson distribution of absolute *MYH7*-mRNA copy numbers in individual cardiomyocytes [90, 91]. Yet, absolute quantification of *MYH7*-mRNA per cell in cardiac tissue from a HCM patient revealed a log-normal distribution which does not support continuous gene expression [82].

During the last decades, evidence increased that most genes are transcribed burst-like; they are switched on and off stochastically at any time [28, 91]. The expression level is determined by the size (the duration) and the frequency of such transcriptional bursts [23]. Burst-like transcription can result in highly heterogeneous gene expression in each cell of seemingly homogeneous cell populations [90, 134]. The independent bursts of transcription of the two alleles may also lead to variable fractions of allelic transcripts and protein from cell to cell [10, 25, 50, 64]. For heterozygous disease-causing mutations, the stochastic transcription of mutated and a wildtype alleles resulting in phenotypic variability from cell to cell may affect severity of certain genetic diseases [25].

**Fig. 4:**
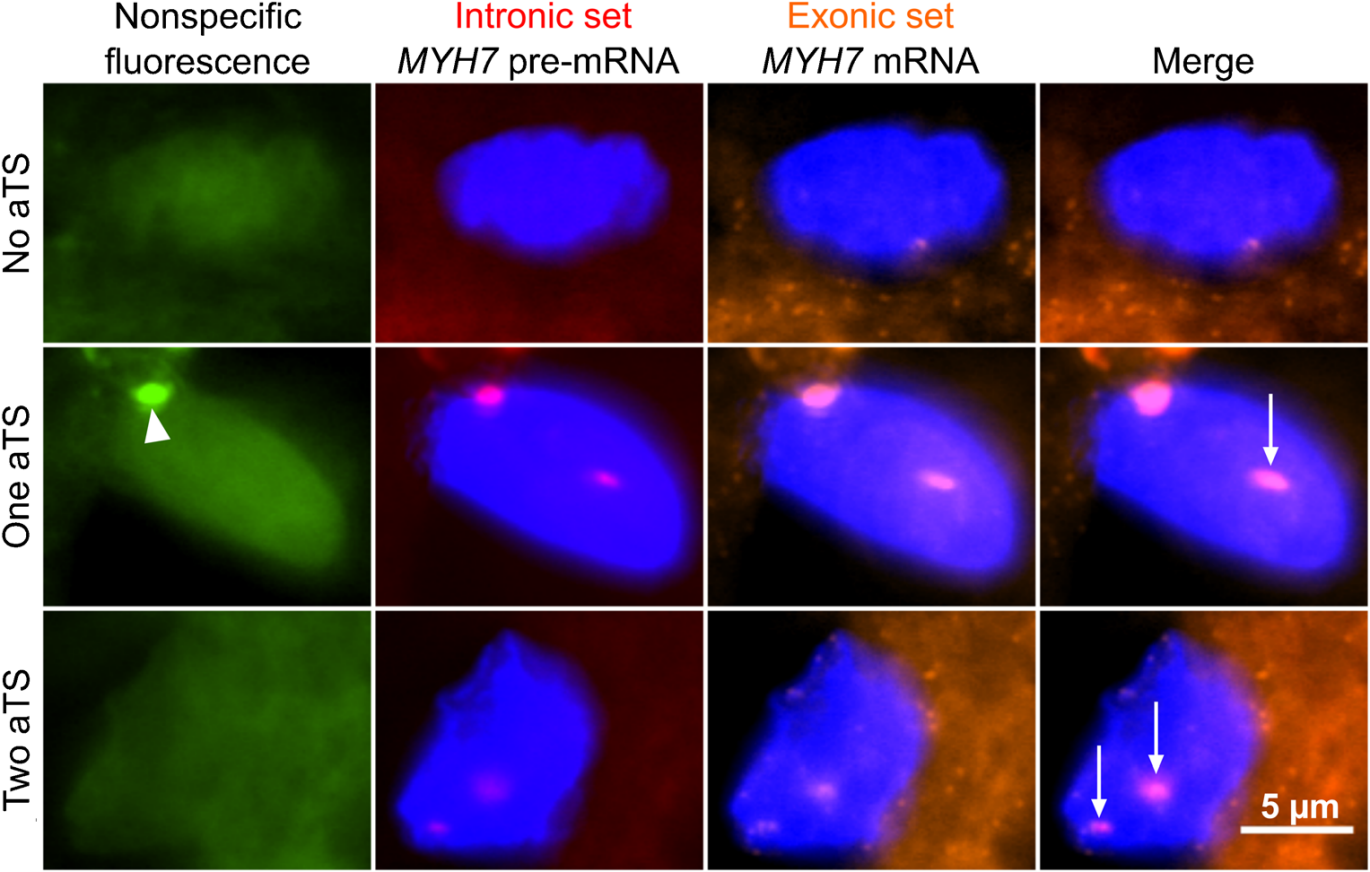
*MYH7* active transcription sites in individual cardiomyocytes of an HCM-patient. Cryo-sections of 16-μm thickness were obtained from cardiac tissue of an HCM patient with the mutation R723G. Fluorescence in situ hybridization (FISH) was performed using an intronic probe set binding the pre-mRNA and an exonic probe set binding the processed mRNA. Co-localization of both fluorescently labeled probe sets in nuclei indicates active transcription sites (aTS). Shown is a cardiomyocyte without aTS (upper panel), a cardiomyocyte with one aTS (middle panel, arrow) and a cardiomyocyte with two aTS (lower panel, arrows). Note that the second signal in the middle panel (arrow head) originates from nonspecific fluorescence (left panel). Figure reprinted from [82] and modified, with permission from Frontiers

We hypothesized that stochastic, burst-like transcription, which is independent for mutant and wildtype *MYH7*-alleles might lead to the observed heterogeneity in *MYH7*-allele expression and function among individual cardiomyocytes from the same patient. To test for burst-like expression, we determined the active transcription sites of the *MYH7*-gene in nuclei of cardiomyocytes of an HCM-patient with mutation R723G by fluorescence in situ hybridization. We found not only nuclei where both alleles were transcribed, but also cells with one active transcription site and, importantly, 27% of the cardiomyocytes were without active transcription sites for *MYH7* (Fig. 4) [82]. This strongly argues against a continuous transcription of the *MYH7*-gene but indicates burst-like transcription [91]. A very recent study confirms this assumption, showing divergent levels of several sarcomeric mRNAs (e.g., *Myh6* and *Myh7)* from cell to cell in rat cardiomyocytes indicating burst-like transcription [67]. In addition, our finding of cardiomyocytes with only one active allele points to the independent activation of both alleles [82]. We assume that burst-like transcription of the two *MYH7*-alleles directly causes the different fractions of wildtype and mutant *MYH7*-mRNA from cell to cell [82]. This conclusion was supported by our mathematical model based on the determined fraction of cells with active transcription sites of the *MYH7*-gene and published rate constants for mRNA-turnover [82].

### Heterogeneous expression and contractile imbalance also for cMyBP-C mutations in HCM

Recent studies suggest that also in patients with cMyBP-C mutations, unequal cMyBP-C-protein abundance from cell to cell exists [88, 110] which may lead to contractile imbalance, thus contributing to HCM pathology [2]. Frameshift mutations in *MYBPC3* usually result in degradation of the truncated protein and lower levels of wildtype cMyBP-C protein, indicating haploinsufficiency [33, 113]. Immunofluorescent or histochemical labelling of cardiac tissue from heterozygous HCM-patients with frameshift cMyBP-C mutations revealed variable distribution of the remaining wildtype cMyBP-C protein among individual cardiomyocytes [2, 88, 110].

**Fig. 5:**
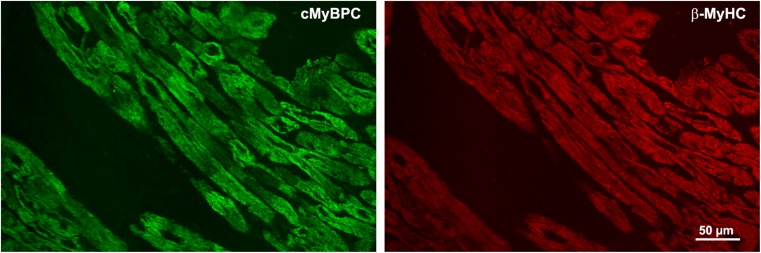
Heterogeneous distribution of cMyBP-C in myocardium of an HCM patient with a truncation mutation. Cryo-sections from cardiac tissue of an HCM-patient with the cMyBP-C-mutation c.927-2A>G were stained with an N-terminus-specific antibody for cMyBP-C (left panel, green) to detect the inter- and intracellular distribution of cMyBP-C. The sections were co-stained with a β-MyHC-specific antibody (right panel, red) to visualize the overall sarcomere fluorescence in the cardiomyocytes. Note the uneven distribution of cMyBP-C between and also within individual cardiomyocytes while the β-MyHC stain is much more regular

Our own studies on cardiomyocytes of a patient with the cMyBP-C-mutation c.927-2A>G, which generates a premature stop-codon between cMyBP-C domains C1 and C2 showed reduced overall cMyBP-C-fluorescence compared to donor cardiomyocytes [2]. Among and within individual cardiomyocytes much more heterogeneous cMyBP-C-fluorescence compared to α-actinin or β-MyHC fluorescent labelling was found (Fig. 5). This suggests unequal abundance of wildtype cMyBP-C protein from cell to cell and patchy distribution within some cardiomyocytes, which might be caused by burst-like transcription of the *MYBPC3*-gene.

Functional studies with the same patient’s cardiomyocytes revealed reduced force generation and higher calcium sensitivity and, interestingly, significantly larger variability of force generation at submaximal calcium levels among *MYBPC3*-mutant cardiomyocytes compared to donor cardiomyocytes [2]. We conclude that the *MYBPC3*-mutation results in variable expression of wildtype cMyBP-C in the patient’s cardiomyocytes, i.e., variable haploinsufficiency (due to allelic imbalance) from cell to cell. This may lead to contractile imbalance, as suggested by the highly heterogeneous force generation at physiological calcium levels, comparable to the heterogeneity we found in the patients with β-MyHC mutations.

### From burst-like transcription to contractile imbalance—possible impact on HCM pathomechanisms

**Fig. 6:**
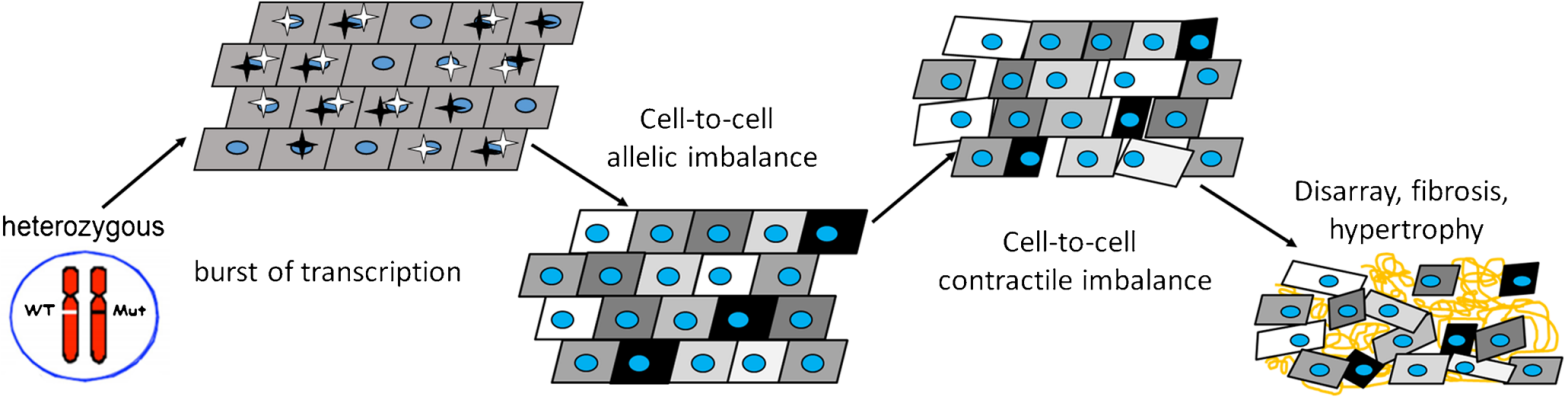
Contractile imbalance hypothesis for *MYH7*-mutations. In heterozygous HCM-patients, both *MYH7* alleles are expressed burst-like; they are switched on and off in an independent and stochastic manner (active mutant and wildtype alleles are indicated by black and white stars). In adult human myocardium, in 27% of nuclei, both alleles were found switched off (no stars, i.e., no active transcription sites in scheme) [82]. Burst-like expression leads to heterogeneous fractions of wildtype and mutant mRNA in neighboring cells (indicated by differently shaded cells). This cell-to-cell allelic mRNA imbalance translates into highly heterogeneous fractions of wildtype and mutant β-MyHC protein among the cells. Due to the effect of the mutations on β-MyHC biomechanical function, the heterogeneous fractions cause imbalance in force generation from cell to cell that disrupts the cardiac syncytium over time. Stronger cells will overstretch weaker cells. This will most likely induce myocyte disarray, fibrosis, and hypertrophy

Based on experimental evidence, our contractile imbalance hypothesis suggests that burst-like transcription of the two *MYH7*-alleles leads to significant variability of mutant and wildtype mRNA fractions among individual cardiomyocytes from heterozygous HCM patients [62, 82]. Most likely, this translates into similar variable fractions of mutant vs. wildtype β-MyHC protein from cell to cell. Since the mutations alter intrinsic β-MyHC-function and thus parameters of force generation of the cardiomyocytes, unequal expression of mutant and wildtype β-MyHC will result in heterogeneous biomechanical properties among individual cardiomyocytes. Over time, this imbalance in the myocardium of HCM patients will presumably contribute to development of cellular disarray and trigger the expression and release of e.g. TGF-β, leading to fibrosis and hypertrophy [17] (Fig. 6). The stochastic nature of burst-like transcription will also lead to changing fractions of mutant and wildtype *MYH7*-mRNA and β-MyHC-protein and thus of force generation per cardiomyocyte over time [62, 82]. This effect may even increase the structural distortions of the myocardium.

Importantly, the contractile imbalance hypothesis is consistent with the poison peptide principle in HCM, stating that the mutated protein is incorporated into the sarcomeres and induces significant alterations of sarcomere contractile function. Evidence suggests that stochastic, independent, burst-like transcription of mutant and wildtype alleles at least for *MYH7* leads to variable fractions of the functionally different wildtype and mutant β-MyHC from cell to cell or even from myofibril to myofibril. Therefore, not only the direct effect of the mutations on β-MyHC structure and function will trigger the development of HCM. Also, contractile imbalance among individual cardiomyocytes [17, 62, 82] and the overall fraction of mutated vs. wildtype protein in the myocardium [7, 26, 35, 39, 81, 86, 111] will contribute to development of HCM. Rare homozygous patients and mouse models show that severely altered function of the myosin molecule itself, its high-dose expression from both alleles, and associated changes in cardiomyocyte physiology can lead to very severe phenotypes which are different from heterozygous patients, as discussed in our previous work [17, 62, 82]. In heterozygous patients, contractile imbalance between cardiomyocytes will exacerbate the mutation-induced development of hallmarks of HCM. Therapeutic interventions in HCM that reduce force generation of the cardiomyocytes with drugs like calcium-channel blockers and β-blockers [17, 62] or small molecule inhibitors like mavacamten will be beneficial since they will reduce contractile imbalance and cardiomyocyte distortions. This is supported by a study on early treatment of HCM in pre-clinical mutation-positive individuals where myocardial remodeling was delayed [137].

The contractile imbalance hypothesis and other current hypotheses on the development of HCM do not necessarily contradict each other. We propose that whenever there is a mutation-induced change in function of the cardiomyocytes (e.g., higher/lower force at the same calcium concentration, altered relaxation) and the affected gene is expressed burst-like with kinetics that result in different fractions of mutant and wildtype protein from cell to cell, then contractile imbalance could develop [17, 62, 82]. Therefore, also if a mutation affects the putative folded-back state of the myosin heads [105] or reduces the number of myosin heads in the super-relaxed state [40], the extent of functional change will be different from cell to cell. It will depend on the respective fractions of mutant and wildtype protein in each cardiomyocyte and thus would lead to contractile imbalance and the associated effects on HCM development.
